# Myopic and Glaucomatous Optic Neuropathy in Highly Myopic Eyes: A Practical Framework for Diagnosis, Monitoring, and Management

**DOI:** 10.3390/jcm15072491

**Published:** 2026-03-24

**Authors:** Masahiro Akada, Shogo Numa, Akitaka Tsujikawa

**Affiliations:** Department of Ophthalmology and Visual Sciences, Kyoto University Graduate School of Medicine, Kyoto 606-8507, Japan

**Keywords:** high myopia, myopic optic neuropathy, glaucoma, optic nerve head, OCT, OCT angiography, visual field, progression, glaucoma surgery

## Abstract

High myopia is increasingly prevalent and complicates glaucoma diagnosis. Axial elongation remodels the optic nerve head (ONH) and parapapillary tissues, producing structural and functional changes that mimic glaucoma—termed myopic optic neuropathy (MON). We reviewed current concepts on the MON–glaucomatous optic neuropathy (GON) spectrum and practical implications for diagnosis, monitoring, and management. A focused PubMed search targeted high/pathologic myopia, glaucoma, ONH and parapapillary anatomy, optical coherence tomography (OCT)/OCT angiography, visual fields, and progression. Major reviews, population-based studies, and longitudinal investigations were prioritized and integrated into a clinician-oriented framework. Greater myopia severity is associated with higher glaucoma risk and, in some cohorts, greater treatment burden, including surgery. Disc tilt, torsion, parapapillary atrophy, and staphyloma-related curvature complicate structural assessment and reduce reliability of single-visit OCT due to magnification and segmentation artifacts. Visual fields may be atypical, and central defects are under-sampled by standard 24-2 testing. Progression-centered strategies—combining event- and trend-based analyses and confirmation rules—distinguish MON-predominant changes from true GON or overlap and guide follow-up. In highly myopic eyes, multimodal structure–function assessment anchored on reproducible progression enhances diagnostic confidence and guides individualized intraocular pressure–lowering therapy. Standardized reporting of myopia definitions and progression criteria is recommended.

## 1. Introduction

Myopia is increasing worldwide and is projected to become an even more prevalent clinical context for optic nerve and visual field (VF) assessment in the coming decades [1]. Beyond introducing measurement bias, myopia is consistently associated with open-angle glaucoma in epidemiologic studies and meta-analyses [2,3,4,5]. Moreover, population-level data suggest that myopia—particularly high myopia—is associated with a greater real-world glaucoma treatment burden, including an increased likelihood of glaucoma surgery [4].

Concurrently, axial elongation induces remodeling of the optic nerve head (ONH), parapapillary tissues, and posterior pole, resulting in disc tilt and torsion, parapapillary atrophy, and staphyloma-related curvature changes. These myopia-associated alterations can produce structural and functional findings that mimic or obscure glaucomatous optic neuropathy (GON), thereby reducing the reliability of single-visit pattern recognition [6,7,8,9].

The term myopic optic neuropathy (MON) is increasingly used to describe optic nerve-related abnormalities primarily attributable to myopic remodeling, whereas GON refers to progressive retinal ganglion cell axonal loss classically associated with glaucoma and is often accompanied by elevated intraocular pressure (IOP) or pressure levels exceeding the individual eye’s tolerance threshold. In clinical practice, MON and GON frequently coexist (MON–GON overlap), and distinguishing MON-predominant changes from true GON or overlap remains a major diagnostic challenge [6,7,8,9].

Prior reviews have extensively addressed the anatomic remodeling and pathophysiologic mechanisms that obscure the distinction between MON and glaucomatous GON, particularly in eyes with high myopia. The present review addresses a complementary, clinician-oriented question: how to determine true structural and functional progression when MON-like abnormalities and measurement artifacts are common at baseline. We synthesize real-world outcome data from large-scale population studies, progression-based principles consistent with contemporary glaucoma guidelines, and myopia-specific limitations of optical coherence tomography (OCT) and standard automated perimetry. To enhance clinical and research applicability, we operationalize these concepts into structured tools: working definitions, a phenotype-based decision matrix, modality-specific pitfall checklists, and a progression-centered monitoring workflow with proposed follow-up intervals and escalation criteria.

## 2. Materials and Methods

We conducted a narrative review of peer-reviewed literature addressing MON and GON in eyes with high myopia. A focused PubMed search was performed from database inception through 14 February 2026 using combinations of the following terms: “high myopia”, “pathologic myopia”, “glaucoma”, “optic neuropathy”, “glaucoma-like optic neuropathy”, “optic nerve head”, “parapapillary atrophy”, “OCT”, “OCTA”, “visual field”, and “progression”. Because this was a narrative review, formal systematic review procedures were not applied; however, we used prespecified relevance principles to guide study selection. Studies were included when they were directly relevant to differentiating MON from GON, particularly regarding optic nerve evaluation, structure–function relationships, imaging interpretation, and progression monitoring. Studies involving pathologic myopia were not automatically excluded when they informed optic nerve assessment or monitoring, although their findings were interpreted with caution when posterior pole abnormalities could confound OCT or visual field outcomes. Studies primarily focused on secondary optic neuropathies or substantial macular comorbidity were generally excluded unless they were directly informative for differential diagnosis or test interpretation.

We prioritized major reviews, population-based studies, and original investigations examining epidemiology, clinical outcomes, diagnostic performance, or longitudinal change in eyes with high myopia. Reference lists of key reviews were also screened to identify additional relevant studies [6,7,8,9].

## 3. Conceptual Framework and Terminology

A practical approach to optic neuropathy in eyes with high myopia requires explicit consideration of two interrelated processes: (i) structural and functional changes driven primarily by axial elongation and myopia-related remodeling (MON-predominant change), and (ii) progressive glaucomatous neurodegeneration (GON). Because these processes may coexist, clinicians should also consider MON–GON overlap, in which both myopic remodeling and true glaucomatous damage contribute to the observed findings [6,7,8,9].

High myopia alters the apparent configuration of the ONH and parapapillary region, including parapapillary zones and scleral and laminar biomechanics [9,10,11,12]. These alterations may displace the retinal nerve fiber layer (RNFL) scan circle, impair segmentation accuracy, and introduce ocular magnification effects that distort thickness measurements. Accordingly, the central diagnostic question is often not “Is this OCT or VF abnormal?” but rather “Is there reproducible, glaucoma-consistent progression?” [6,7,8,9,13,14,15].

Table 1 provides the working definitions used throughout this review. Table 2 operationalizes a progression-centered, structure–function-integrated framework that clinicians may apply in practice and researchers may reference when defining phenotypes and outcomes.

## 4. Epidemiology and Clinical Burden: From Association to Outcomes

Beyond the association between myopia and glaucoma, epidemiologic interpretation is complicated by the fact that the population-based prevalence and incidence of MON and MON–GON overlap remain poorly standardized. High myopia itself is becoming increasingly prevalent worldwide, and this rising denominator is expected to increase the clinical frequency of optic nerve assessment in myopic eyes. However, direct estimation of MON prevalence or MON–GON overlap remains challenging because definitions vary across studies and because structural myopic remodeling and glaucomatous change often coexist along a spectrum rather than as mutually exclusive categories.

Epidemiologic studies have consistently reported an association between myopia and open-angle glaucoma, and meta-analyses support a dose–response relationship in which increasing degrees of myopia are associated with progressively higher glaucoma risk [2]. Population-based cohorts, including the Blue Mountains Eye Study and the Beijing Eye Study, provide foundational evidence supporting this association [3,5].

An important clinical implication is that high myopia functions not only as a diagnostic confounder but also as a potential contributor to clinically meaningful outcomes. In a nationwide Japanese cohort study including more than 14 million adults aged ≥40 years, myopia and high myopia were associated with higher hazards of incident glaucoma and glaucoma surgery, with particularly elevated hazards for filtering surgery among individuals with high myopia [4]. These findings support intensified surveillance and timely IOP—lowering intervention when longitudinal evidence demonstrates GON or MON–GON overlap.

Table 3 summarizes modality-specific pitfalls that may lead to diagnostic misclassification. Table 4 highlights selected high-impact studies and reviews frequently cited for epidemiology, diagnostic frameworks, and myopia-specific measurement limitations.

These pitfalls affect more than baseline classification. Mislabeling MON-predominant change as glaucoma may lead to unnecessary treatment escalation and overinterpretation of artifact-prone imaging, whereas mislabeling true GON as myopic change may delay central testing and increase the risk of missed progression. For this reason, the diagnostic pitfalls summarized in Table 3 should directly inform surveillance intensity, modality selection, and the threshold for confirming change in the workflow outlined in Table 5.

## 5. Structural Assessment in Eyes with High Myopia

### 5.1. Optic Disc and Parapapillary Phenotyping

Clinical examination of the optic disc in eyes with high myopia should begin with systematic phenotype documentation rather than immediate categorization as glaucomatous or nonglaucomatous. Disc tilt and torsion, parapapillary atrophy, parapapillary intrachoroidal cavitation, and posterior staphyloma can alter the apparent neuroretinal rim configuration and modify the distribution of RNFL bundles [8,9,10,11,12,39]. Because these alterations may be asymmetric, careful inter-eye comparison and longitudinal photographic and OCT documentation are particularly valuable.

A practical strategy is to describe (i) disc insertion characteristics and torsion axis, (ii) the extent and subtype of parapapillary atrophy or zones, and (iii) posterior pole curvature features, and then interpret OCT and perimetry findings within that phenotypic context [9,10,11,12,39]. Data-driven subclassification of glaucomatous eyes with high myopia based on disc tilt and parapapillary features further supports this phenotype-first approach and demonstrates that distinct phenotypes may exhibit characteristic VF defect patterns [22].

### 5.2. OCT RNFL and Macular Ganglion Cell Metrics

OCT-derived RNFL thickness is widely used for glaucoma detection; however, in eyes with high myopia, it is susceptible to both false-positive and false-negative interpretations. True myopia-related remodeling and imperfect alignment with normative databases may yield sectors categorized as “outside normal limits”, even in nonglaucomatous myopic eyes. In addition, ocular magnification, disc tilt, scan circle decentration, and segmentation errors can further distort circumpapillary RNFL maps [13,16,17]. Accordingly, clinicians should routinely review B-scans and segmentation boundaries, confirm scan centration, and treat color-coded classifications as hypothesis-generating rather than definitive [13,16]. When circle- or map-based summaries are unreliable, a targeted vertical B-scan to sample the papillomacular bundle can be a practical adjunct for assessing the integrity of central axonal pathways and contextualizing central VF findings.

Macular ganglion cell layer and inner plexiform layer (ganglion cell layer or ganglion cell–inner plexiform layer) metrics may enhance structure–function correlation in some glaucomatous eyes; however, high myopia frequently coexists with macular pathology, including myopic macular degeneration, tractional changes, and myopic macular neovascularization. These comorbidities may confound macular thickness-based interpretation [16]. Population-level evidence indicates that myopic macular neovascularization represents a clinically significant macular comorbidity in high myopia, reinforcing the importance of documenting macular status when interpreting macular OCT findings and central VF defects in suspected MON–GON overlap [18].

### 5.3. Bruch’s Membrane Opening- and Parapapillary Zone-Aware Interpretation

A recurring principle in the evaluation of glaucoma in eyes with high myopia is that conventional optic disc margin assumptions may be unreliable when Bruch’s membrane opening (BMO) position and parapapillary zones shift with axial elongation. Histology-informed syntheses and imaging studies indicate that parapapillary zones (alpha, beta, gamma, and delta) and BMO geometry reflect axial elongation and may alter the apparent relationship between neuronal rim tissue and the RNFL [9,10,11,12]. Longitudinal OCT studies further demonstrate that parapapillary structures can evolve over years in adult eyes with high myopia, underscoring the importance of serial scan comparison [12]. In highly myopic POAG eyes, swept-source OCT studies have linked focal lamina cribrosa defects to myopic disc morphology and parapapillary features such as PPA without Bruch’s membrane and peripapillary intrachoroidal cavitation, supporting phenotype-aware parapapillary interpretation [40].

### 5.4. OCT Angiography as a Supportive Tool

OCT angiography (OCTA) may provide complementary information regarding parapapillary and juxtapapillary microvasculature; however, high myopia increases susceptibility to motion and segmentation artifacts and may alter vascular density metrics. In glaucoma cohorts, lower parapapillary choroidal vessel density has been associated with subsequent VF progression, and central VF damage has been linked to choroidal microvasculature dropout, including in eyes with high axial myopia [19,20]. In clinical practice, OCTA findings should be interpreted as corroborative evidence that must be reproducible and concordant with the overall MON–GON phenotype.

## 6. Functional Assessment: VF Testing in High Myopia

VF interpretation is central to differentiating MON from GON; however, high myopia is associated with characteristic nonglaucomatous VF patterns and greater test–retest variability. Classification studies of eyes with high myopia without pathologic myopic maculopathy have described recurrent VF abnormality patterns that can mimic glaucoma, underscoring the need for caution when diagnosing glaucoma on the basis of a single VF examination [23].

### 6.1. Common Myopia-Related VF Patterns and Their Pitfalls

Myopia-related VF defects include an enlarged blind spot, temporal defects associated with disc tilt or torsion, and localized defects influenced by parapapillary atrophy or staphyloma-related posterior pole curvature. In myopic glaucoma, temporal VF defects have been characterized and may demonstrate distinct progression trajectories [26]. The direction and magnitude of parapapillary tilt can influence central VF vulnerability in primary open-angle glaucoma with high myopia, reinforcing the need to interpret VF defect location within the context of ONH phenotype [27].

### 6.2. Central VF Vulnerability and the Role of 10-2/24-2C Testing

Central VF loss may be clinically consequential in highly myopic eyes, particularly when structural changes involve the posterior pole. Posterior pole curvature alterations and scleral deformation around the ONH have been linked to central visual function, and ONH curvature flattening has been associated with central VF scotoma in myopic eyes [24,41]. In clinical practice, incorporating 10-2 testing (or a 24-2C strategy, when available) can improve detection and monitoring of central damage. Standard 24-2 testing may underestimate central defects and macular progression that are more sensitively detected by central strategies interpreted in conjunction with macular OCT metrics [30,31,42]. In a multicenter study of advanced open-angle glaucoma (MD < −15 dB), higher myopic refractive error was associated with greater damage near fixation (lower cecocentral region) in eyes with high IOP, and the authors concluded that high myopia increases vulnerability of the remaining lower cecocentral VF in advanced glaucoma with elevated IOP [21].

From a practical standpoint, standard 24-2 testing remains useful for broad-field surveillance and continuity with conventional glaucoma monitoring, but it undersamples the central 10 degrees. A 24-2C strategy may be useful when clinicians wish to retain continuity with 24-2-based monitoring while improving central sampling. In contrast, 10-2 testing is particularly helpful when paracentral or fixation-threatening damage is suspected, when macular OCT findings raise concern for central structure–function loss, or when symptoms and 24-2 findings are discordant. In highly myopic eyes, adding a central strategy should be considered when central involvement is clinically suspected or when central structure–function interpretation is especially important, rather than relying on 24-2 alone.

### 6.3. Longitudinal VF Change: Distinguishing Progression from Variability

Because cross-sectional VF abnormalities are common in high myopia, longitudinal confirmation is often decisive. Long-term follow-up studies of myopic glaucoma have reported progression rates and associated risk factors, and prediction studies in nonpathologic high myopia have identified baseline features associated with incident VF defects [28,29]. From a practical perspective, clinicians should prioritize reproducible trend-based change across serial examinations and seek stable structure–function concordance over time (Table 2) [6,7,8,9,23,26,27,28,29].

## 7. Progression-Centered Diagnosis and Monitoring in the MON–GON Spectrum

### 7.1. Why Progression Is the Key Discriminator

In eyes with high myopia, cross-sectional abnormalities on optic disc examination, OCT, and standard automated perimetry are common and may reflect MON-predominant remodeling rather than true GON. This creates a practical paradox: the more abnormal the baseline structure or function appears, the less certain the diagnosis of glaucoma. A progression-centered framework helps resolve this paradox by prioritizing reproducible longitudinal worsening as the primary trigger for diagnostic reclassification and therapeutic escalation. This approach is consistent with guideline-based glaucoma management, in which treatment intensity and target IOP are adjusted according to the observed disease trajectory over time rather than isolated baseline findings [34,35]. Within the MON–GON spectrum, this strategy seeks to reduce the overtreatment of stable MON phenotypes while minimizing the undertreatment of genuinely progressive GON or MON–GON overlap.

### 7.2. Operationalizing Progression: Event-Based, Trend-Based, and Confirmation Rules

Progression can be conceptualized as event-based (change exceeding expected test–retest variability relative to baseline) or trend-based (estimation of a rate of change over time), and integrative strategies that combine both paradigms may enhance detection accuracy and clinical decision-making [32,33]. Event-based tools, such as Guided Progression Analysis, may be sensitive to early change; however, their specificity is influenced by measurement variability. False-positive progression signals are more likely in patients with greater fluctuation, reinforcing the need for conservative confirmation criteria and meticulous artifact review [43]. Trend-based approaches provide a quantitative estimate of velocity (e.g., slope of mean deviation or visual field index, or pointwise linear regression) that supports risk stratification, follow-up interval planning, and communication with patients [32,33].

In eyes with high myopia, regardless of the analytic paradigm used, a practical evaluation criterion is to require reproducibility, defined as a similar direction and topographic distribution of change on consecutive examinations. Clinicians should explicitly exclude artifacts (e.g., segmentation failure, scan decentration, media opacity, poor fixation) and nonglaucomatous explanations (e.g., active macular disease, cataract progression, ocular surface disease) before declaring progression [16,33].

In practical terms, repeatable progression in highly myopic eyes should generally require more than a single abnormal test and should be supported by the following elements: a consistent direction of change, a similar topographic pattern across repeat examinations, and recurrence on consecutive testing after the exclusion of artifactual and nonglaucomatous explanations. For event-based visual field analyses, at least two reliable baseline fields are typically required, and repeat change on consecutive follow-up tests provides greater confidence than an isolated event. For trend-based endpoints such as MD or VFI slopes, isolated pairwise comparisons are insufficient, and interpretation should rely on multiple serial examinations over time. Because local and fluctuation-prone endpoints are more vulnerable to false-positive signals, pointwise or cluster-based analyses generally warrant stricter confirmation than global trend measures.

### 7.3. Structural Progression: Using OCT Longitudinally in High Myopia

OCT is indispensable for longitudinal follow-up; however, high myopia amplifies common failure modes that can mimic structural progression. Apparent RNFL thinning may result from segmentation errors near parapapillary atrophy, scan-circle decentration in tilted discs, magnification-related sampling differences, or inconsistent image quality across visits. Accordingly, longitudinal interpretation should routinely include: (i) direct inspection of B-scans and layer boundaries; (ii) use of consistent scan protocols and device-specific follow-up modes, when available; and (iii) confirmation that the spatial pattern of change is biologically plausible and topographically coherent over time [16]. Because OCT artifacts are frequent in eyes with high myopia, color coding and deviation maps should be treated as hypothesis-generating rather than diagnostic [13]. Where available, serial comparison of a standardized papillomacular bundle-oriented line scan can further support the adjudication of central structural change.

When circumpapillary RNFL metrics are unstable, complementary approaches—including macular ganglion cell or inner plexiform layer analyses, wide-field imaging strategies, and standardized optic disc documentation—can strengthen longitudinal inference, particularly when suspected damage involves the macula or papillomacular bundle [16]. Deep ONH structural parameters, such as lamina cribrosa curvature or configuration, have been associated with subsequent VF progression in primary open-angle glaucoma and may help contextualize structural change; however, their clinical integration in highly myopic phenotypes requires further standardization and validation [44]. Three-dimensional swept-source OCT has also enabled the direct visualization of focal lamina cribrosa defects in glaucoma and their associations with clinical features such as disc hemorrhage, providing a potential anatomic target for phenotype-based monitoring even in highly myopic eyes [45].

### 7.4. Functional Progression: Central Testing, Reproducibility, and Macular Confounding

Central VF loss is clinically consequential; however, standard 24-2 strategies undersample the central 10 degrees. In glaucoma suspects and in eyes with early glaucoma, clinically meaningful central defects detected by 10-2 testing are frequently missed on 24-2, supporting the use of 10-2 assessment when central involvement is suspected or when macular structural abnormalities are present [30].

Macular progression may also be underestimated by 24-2-based progression algorithms, reinforcing the rationale for central functional assessment aligned with macular structural monitoring [31]. For longitudinal evaluation, reproducibility should be prioritized: repeat testing early to mitigate learning effects, interpret changes in the context of reliability indices, and consider pointwise or cluster-based analyses when global indices are insensitive [32,33]. In highly myopic eyes, it is essential to distinguish glaucoma-consistent change from macular-driven sensitivity loss (e.g., myopic macular degeneration, tractional pathology, or scarring secondary to myopic macular neovascularization), and to document these confounders alongside VF interpretation [16,18]. A structured classification of VF abnormality phenotypes in high myopia may facilitate communication and reduce the misclassification of MON-predominant patterns as definitive glaucoma at baseline [23]. In early glaucoma with high myopia, circumpapillary OCTA vessel density shows meaningful structure–function relationships and can complement cpRNFL when myopic anatomy complicates structural interpretation.

### 7.5. When Progression Is Uncertain: Escalate Measurement, Not Assumptions

Structure–function discordance is common in eyes with high myopia. For example, myopia-related peripapillary entities such as peripapillary intrachoroidal cavitation may not consistently produce corresponding field loss, supporting conservative confirmation rules before labeling change as glaucomatous progression [36]. When change is suspected but not yet confirmed, a defensible strategy is to intensify and standardize measurements—for example, repeat OCT and VF testing within 3–6 months using identical acquisition protocols—while explicitly documenting potential confounders (e.g., cataract status, ocular surface disease, macular disease activity, treatment modifications) [16,33]. A short interval of closer surveillance often clarifies whether change is reproducible and topographically coherent.

In higher-risk phenotypes, clinicians may consider interim risk-mitigation measures, including IOP lowering, while anchoring major therapeutic escalation decisions to documented progression whenever feasible, consistent with guideline-based glaucoma management [34,35]. In addition, systemic or behavioral factors associated with neuroretinal vulnerability (e.g., sleep disturbance) may be incorporated into individualized risk profiling and follow-up interval planning [25].

Given population-based evidence that myopia and high myopia are associated with higher hazards of incident glaucoma and glaucoma surgery, a higher pretest probability and lower tolerance for delayed confirmation may be reasonable in selected patients, particularly when longitudinal signals suggest true GON or MON–GON overlap [4]. Table 5 provides a pragmatic, progression-centered workflow that can be adapted to local practice.

### 7.6. Why the Evidence on Myopia and Glaucoma Progression Is Heterogeneous (And How to Interpret It)

The literature evaluating whether myopia accelerates, has no measurable effect on, or appears to attenuate VF progression is heterogeneous. Rather than interpreting these findings as purely biological contradictions, clinicians and researchers should recognize recurring methodological drivers of heterogeneity: (i) inconsistent definitions of myopia (spherical equivalent vs. axial length, and inclusion or exclusion of pathologic myopia); (ii) baseline disease severity and floor effects that influence apparent rates of change; (iii) confounding by indication (more aggressive treatment in eyes perceived to be at higher risk); (iv) differences in perimetry strategy and sensitivity to central change (24-2 vs. 10-2 or hybrid approaches); and (v) myopia-specific artifacts and macular comorbidity that distort both OCT and VF endpoints [16,32,33].

For example, some cohorts report no association between myopia and VF progression and suggest that high myopia may be protective for photographic optic disc or RNFL progression; such findings may partly reflect difficulty in detecting disc or RNFL change or misclassification at baseline [46]. Conversely, studies in highly myopic primary open-angle glaucoma emphasize that the observed clinical course depends on the relative dominance of GON versus MON remodeling within the same diagnostic category, supporting the MON–GON mixture concept [47]. Recent analyses leveraging dense VF series further suggest region-specific effects of myopia on VF loss and progression that vary by myopia severity and analytic endpoint [48].

Accordingly, the question of “myopia versus progression” should not be reduced to a single headline effect size. Instead, studies should be interpreted—and designed—with the transparent reporting of baseline stage, longitudinal treatment exposure, axial length, macular comorbidity, and explicitly defined progression criteria with confirmation rules. Such standardization would improve comparability and facilitate future meta-analytic synthesis. To enhance reproducibility and comparability, we propose a minimum reporting set for MON–GON studies and clinical series (Table 6). Table 6 is intended not only as a minimum reporting set, but also as a practical appraisal framework for evaluating the comparability, external validity, and interpretability of future MON–GON studies. Readers should interpret external validity more cautiously when studies rely on non-myopic normative databases without adjustment, do not report axial length or macular status, discuss central outcomes on the basis of 24-2 testing alone, or do not clearly define progression confirmation rules.

## 8. Management Considerations in Highly Myopic Eyes

### 8.1. When to Initiate IOP–Lowering Therapy

Given the high prevalence of MON-like structural and functional abnormalities in eyes with high myopia, initiation of long-term IOP-lowering therapy is best justified by one of the following: (i) confirmed progression consistent with glaucoma on VF testing and/or OCT; (ii) strong, reproducible structure–function concordance consistent with a glaucomatous pattern; or (iii) a high-risk clinical context in which the pretest probability of GON is sufficiently elevated to justify treatment despite residual diagnostic uncertainty [6,7,8,9,23,26,27,28,29].

Conversely, stable phenotypes with poor structure–function concordance—particularly in younger patients—should prompt caution to avoid overtreatment while ensuring structured longitudinal follow-up to detect evolution toward MON–GON overlap (Table 2 and Table 5) [8].

In eyes with relatively severe VF loss but a predominantly MON phenotype and no reproducible progression, routine long-term IOP-lowering treatment is not directly supported by MON-specific evidence. In such cases, careful observation with intensified longitudinal confirmation is generally preferred, particularly when the clinical picture remains dominated by myopic structural change. However, IOP-lowering treatment may still be considered when glaucoma risk is otherwise high, such as in the presence of consistently elevated or markedly fluctuating IOP, disc hemorrhage, thin central corneal thickness or low corneal hysteresis, strong family history, fellow-eye definite glaucoma, or concordant structure–function changes threatening fixation.

### 8.2. Setting Targets and Selecting Interventions

When GON is suspected or confirmed, target IOP should be individualized according to baseline structural and functional damage, documented rate of progression, and patient-specific risk tolerance. Nationwide cohort data demonstrating that high myopia is associated with increased risks of glaucoma surgery underscore the potential clinical burden and support appropriately intensive medical therapy when progression is established [4].

Surgical decision-making in highly myopic eyes requires individualized counseling and careful postoperative monitoring, as particular posterior pole anatomy and macular comorbidities may influence functional outcomes. Clinicians should document macular status and central visual function preoperatively and incorporate central VF monitoring into postoperative surveillance when relevant [18,24,41]. In myopic normal-tension glaucoma, trabeculectomy has been reported to reduce IOP and to slow central (10-2) visual field deterioration, underscoring the clinical relevance of central functional monitoring when escalation to surgery is considered [38].

## 9. Research Gaps and a Proposed Minimum Reporting Set

Despite advances in imaging and analytic methodologies, several limitations continue to constrain progress in the MON–GON field. First, normative databases and diagnostic thresholds remain suboptimal for highly myopic eyes, and magnification and segmentation artifacts remain pervasive in clinical imaging [13]. Second, definitions of “high myopia”, MON, and MON–GON overlap vary across studies, complicating evidence synthesis and clinical translation [6,7,8,9]. Third, macular comorbidity is common in highly myopic eyes and can distort both structural and functional endpoints; however, reporting of macular status and disease activity is inconsistent [18]. For example, a highly myopic macular ganglion cell complex normative database substantially improved specificity for detecting early glaucoma compared with a non-myopic database in a highly myopic cohort [49]. Additionally, macular inner retinal layer thickness asymmetry indices derived from vertical macular B-scans have been proposed as sensitive indicators of glaucomatous damage [50]. Such asymmetry-based approaches may be attractive when absolute thickness metrics are confounded by myopic anatomy.

To improve comparability and facilitate future meta-analyses, Table 6 proposes a pragmatic minimum reporting set for clinical studies of optic neuropathy in highly myopic eyes. This proposed framework is intended to support standardized reporting in observational studies, interventional trials, and diagnostic accuracy research.

## 10. Conclusions

High myopia is increasingly prevalent and is associated with a higher risk of glaucoma and glaucoma surgery, indicating that its clinical burden extends beyond diagnostic uncertainty [1,2,3,4,5]. Because myopia-related ONH remodeling can mimic or obscure glaucomatous damage, clinicians should adopt a MON–GON spectrum framework and prioritize longitudinal, reproducible progression supported by structure–function concordance rather than single-visit abnormalities [6,7,8,9,13,14,15].

A structured workflow—including phenotype documentation, rigorous evaluation of OCT image quality, explicit assessment of macular confounders, and strategic perimetry with central VF testing when indicated—may improve diagnostic confidence and support timely, individualized IOP-lowering management when GON is suspected or confirmed (Table 1, Table 2, Table 3, Table 4 and Table 5).

## Figures and Tables

**Table 1:** Working definitions and terminology for MON and GON in highly myopic eyes.

Term	Working Definition	Notes for Clinicians
High myopia	Myopia associated with myopia-related structural remodeling; commonly defined as spherical equivalent ≤ −6.0 D and/or axial length ≥ 26.0 mm.	Use locally defined cutoffs; record axial length when available.
Myopic optic neuropathy (MON)	Optic nerve structural or functional abnormality primarily attributable to myopic remodeling of the ONH and parapapillary tissues; considered largely IOP-independent, although a contributory role cannot be excluded.	May be static or slowly progressive; “glaucoma-like” does not equal glaucoma.
Glaucomatous optic neuropathy (GON)	Progressive retinal ganglion cell axon loss consistent with glaucoma, often with typical structure–function concordance.	May coexist with MON; documented progression is critical.
MON–GON overlap	Coexistence of myopic remodeling and glaucomatous progression in the same eye.	Often the most clinically challenging category.
Structure–function concordance	Spatial agreement between structural loss (optic disc/RNFL/GCL) and visual field defects.	Strong concordance increases the likelihood of glaucoma in highly myopic eyes.
Progression-centered diagnosis	Diagnostic approach that prioritizes reproducible longitudinal change over cross-sectional “outside normal limits” classifications.	Particularly important in highly myopic eyes.

Abbreviations: GCL, ganglion cell layer; IOP, intraocular pressure; ONH, optic nerve head; RNFL, retinal nerve fiber layer. Note: Because included studies variably defined high myopia using spherical equivalent, axial length, or both, we interpreted each study within its original definitional framework and avoided overstandardizing heterogeneous source literature.

**Table 2:** Decision matrix for interpreting MON-predominant versus GON-predominant/overlap presentations.

Clinical Question	MON-Predominant More Likely When…	GON-Predominant/Overlap More Likely When…
Is the phenotype stable?	VF or OCT abnormalities remain unchanged across multiple visits with consistent test quality.	Trend-based worsening on VF testing and/or OCT exceeding expected test–retest variability.
Do findings correspond structurally and functionally?	Poor or inconsistent spatial correspondence between structural and functional findings.	Clear structure–function concordance (e.g., localized RNFL/GCL loss corresponding to a VF defect cluster).
How reliable are OCT metrics?	Marked disc tilt, PPA, or staphyloma associated with frequent segmentation or centration artifacts; color maps vary with scan placement.	High-quality scans with stable segmentation; consistent focal loss over time.
Are alternative causes of VF loss present?	Coexisting myopic macular disease, traction, scarring plausibly explains VF defects.	Macula relatively preserved; defects follow glaucoma-consistent topology.
What is the baseline risk?	Younger patients with low systemic risk and low post-evaluation suspicion after comprehensive assessment.	High myopia with additional glaucoma risk factors; epidemiologic data indicate increased hazard of glaucoma and need for surgery.

Abbreviations: GCL, ganglion cell layer; OCT, optical coherence tomography; PPA, parapapillary atrophy; RNFL, retinal nerve fiber layer; VF, visual field.

**Table 3:** Common diagnostic pitfalls in highly myopic eyes and practical mitigation strategies.

Modality	Common Pitfall in High Myopia	Practical Mitigation	Key References
OCT RNFL	Normative database mismatch, scan circle placement variability, and segmentation errors near PPA may create apparent RNFL abnormalities.	Inspect B-scans, document scan quality, avoid overreliance on color-coded maps, compare like-with-like longitudinally before attributing apparent thinning to true progression.	[13,16,17]
Macular GCL/GCIPL	Macular pathology or traction confounds ganglion cell metrics and central functional interpretation, including changes related to myopic macular degeneration or scarring after mMNV.	Correlate with macular OCT and consider central perimetry when macular metrics are suspicious; focal central sensitivity loss that corresponds to macular pathology but does not follow a typical arcuate glaucomatous pattern should be interpreted cautiously.	[16,17,18]
BMO/ONH metrics	BMO shift and parapapillary zones distort disc margin assumptions and may produce apparent neuroretinal rim abnormalities that are difficult to compare across visits.	Use multimodal imaging, interpret rim parameters cautiously, and prioritize longitudinal changes over single-visit threshold deviations.	[9,10,11,12]
OCTA	Motion and projection artifacts, segmentation errors in elongated eyes, and unstable vessel density measurements may mimic or exaggerate disease-related change.	Treat as supportive only; confirm reproducibility and ensure structure–function alignment before attributing apparent vascular change to true progression.	[19,20]

Abbreviations: OCT, optical coherence tomography; RNFL, retinal nerve fiber layer; GCL, ganglion cell layer; GCIPL, ganglion cell layer/inner plexiform layer; BMO, Bruch’s membrane opening; ONH, optic nerve head; OCTA, OCT angiography; PPA, parapapillary atrophy.

**Table 4:** Selected studies and reviews relevant to MON and GON in high myopia (epidemiology, diagnostic framework, and measurement limitations).

Study	Design/Population	Main Outcome	Key Findings
Holden et al., Ophthalmology 2016 [1]	Global systematic modeling of refractive error prevalence	Projected myopia and high myopia burden	Projects ~50% of the world population to be myopic and ~10% highly myopic by 2050.
Marcus et al., Ophthalmology 2011 [2]	Systematic review/meta-analysis of observational studies	Association between myopia and open-angle glaucoma	Pooled OR ~1.9 for myopia; higher odds with greater myopia severity.
Mitchell et al., Ophthalmology 1999 (Blue Mountains Eye Study) [3]	Population-based cross-sectional study	Relationship between glaucoma and myopia	Supports an association between myopia and glaucoma in population data.
Akada et al., Ophthalmology 2026 (nationwide cohort, Japan) [4]	Nationwide longitudinal cohort (>14 million adults aged ≥40 years)	Incident glaucoma; glaucoma surgery	Adjusted HR for incident glaucoma: 1.44 (myopia) and 2.67 (high myopia). Adjusted HR for glaucoma surgery: 1.71 and 3.07; filtering surgery: 2.03 and 4.03.
Xu et al., Ophthalmology 2007 (Beijing Eye Study) [5]	Population-based study	High myopia and glaucoma susceptibility	Increased glaucoma susceptibility in highly myopic eyes in a large population cohort.
Zhang et al., Prog Retin Eye Res 2024 [6]	Comprehensive review	Optic neuropathy in high myopia (glaucoma vs. myopia vs. both)	Synthesizes overlapping phenotypes and emphasizes longitudinal confirmation and multimodal evaluation.
Jiravarnsirikul et al., Surv Ophthalmol 2025 [7]	Comprehensive review	Practical evaluation of glaucoma in myopic eyes	Summarizes performance and limitations of OCT, OCTA, and perimetry in myopic eyes.
Jonas et al., Asia Pac J Ophthalmol 2020 [8]	Clinical review	Glaucoma-like optic neuropathy in high myopia	Describes structural changes in high myopia resembling glaucoma and proposes discriminating features.
Wang et al., Prog Retin Eye Res 2021 [9]	Histology/clinical synthesis	Parapapillary zones (alpha/beta/gamma/delta) in myopia and glaucoma	Clarifies anatomy and clinical implications of parapapillary zones for axial elongation and glaucoma assessment.
Poon et al., J Glaucoma 2023 [13]	Clinical study	Prevalence of OCT artifacts in high myopia	OCT artifacts are common and can materially influence glaucoma diagnosis.
Akada et al., Ophthalmol Sci 2025 [18]	Nationwide claims cohort	Incidence and patterns of mMNV	Highlights population-level mMNV burden and treatment patterns in high myopia.
Mayama et al., Ophthalmology 2002 [21]	Multicenter cross-sectional (advanced OAG)	In advanced OAG (MD < −15 dB), higher myopia worsens lower cecocentral VF damage.	Reinforces central VF vigilance and the rationale for 10-2/24-2C testing when high myopia coexists with suspected/confirmed glaucoma.
Han et al., PLoS ONE 2017 [22]	Cluster analysis/phenotyping	Myopic glaucomatous eyes (AXL ≥ 24 mm; includes moderate myopia) clustered into two phenotypes primarily distinguished by optic disc tilt and β-zone PPA width; VF defect locations differed between clusters.	Supports phenotype-first documentation and helps contextualize heterogeneity in structure–function patterns within the MON–GON spectrum.
Lin et al., Ophthalmology 2022 [23]	Classification study in non-pathologic high myopia	Patterns of VF abnormalities	Defines and categorizes nonglaucomatous myopia-related VF patterns.
Park et al., J Clin Med 2024 [24]	Imaging-function association	ONH curvature flattening and central VF scotoma	ONH curvature correlates with central VF scotoma, supporting biomechanical contribution to central VF vulnerability.
Akada et al., Am J Ophthalmol 2025 [25]	Community OCT+nationwide cohort	Sleep parameters/disorders; RNFL thinning; incident glaucoma	Sleep insufficiency and clinically diagnosed sleep disorders were associated with thinner RNFL and higher glaucoma risk.

Abbreviations: MON, myopic optic neuropathy; GON, glaucomatous optic neuropathy; OCT, optical coherence tomography; OCTA, OCT angiography; VF, visual field; MD, mean deviation; RNFL, retinal nerve fiber layer; PPA, parapapillary atrophy; ONH, optic nerve head; mMNV, myopic macular neovascularization; OAG, open-angle glaucoma.

**Table 5:** Progression-centered diagnosis and monitoring workflow for MON–GON phenotypes in highly myopic eyes.

Workflow Step	Core Actions (High-Myopia Specific)	Practical Adjudication/Escalation Trigger	Key References
(1) Baseline phenotype & confounders	Refractive error/axial length; posterior staphyloma if known Disc/peripapillary phenotype (tilt/torsion, PPA, parapapillary zones) Macular status (myopic maculopathy, traction, mMNV) IOP profile and systemic/contextual risks	Define pretest probability (MON-predominant vs. GON-predominant/overlap) and set monitoring intensity accordingly; document confounders that may mimic progression.	[6,7,8,9,18,23,26,27,28,29]
(2) Build a reliable baseline	OCT (cpRNFL+macular GCL/GCIPL) with saved B-scans; consistent protocol Standard automated perimetry: 24-2/30-2; consider 10-2/24-2C when central involvement suspected Disc photographs/multimodal imaging when available	Avoid major escalation based on a single abnormal test; obtain repeatable baseline tests to anchor event- and trend-based analyses.	[16,30,31,32,33]
(3) Risk-stratified follow-up cadence	MON-predominant/low suspicion: OCT+VF ~6–12 months Indeterminate/suspect: repeat OCT+VF in ~3–6 months Confirmed/high-risk overlap: VF more frequently; consider central VF strategy	Shorten intervals when variability is high, central function threatened, or slopes are steep; reassess after enough tests to estimate a rate of change.	[30,31,33,34,35]
(4) Structural progression (OCT)	Inspect raw B-scans and segmentation lines Check scan centration and artifacts near PPA/staphyloma If cpRNFL unstable, emphasize macular GCL/GCIPL and disc documentation	Declare structural progression only if reproducible, topographically coherent, and not explained by scan artifacts or macular pathology.	[13,16,17]
(5) Functional progression (VF) and myopia-specific mimics	Prioritize reproducibility; repeat early when change is suspected Use 10-2/24-2C if central loss suspected or macular metrics abnormal Document macular/peripapillary confounders (e.g., mMNV scars; PICC)	Confirm functional progression with consistent topography across tests; avoid attributing sensitivity loss to GON without excluding macular/peripapillary causes.	[18,30,31,36]
(6) Integrate structure–function and decide escalation	Evaluate concordance between OCT/OCTA and VF within the same anatomic framework When discordant, intensify measurement rather than assumptions (repeat OCT/VF with identical protocols)	Escalate IOP-lowering intensity primarily when reproducible progression is documented; consider surgical escalation when targets cannot be met or progression continues.	[32,33,34,35,37,38]

Abbreviations: OCT, optical coherence tomography; VF, visual field; cpRNFL, circumpapillary retinal nerve fiber layer; GCL, ganglion cell layer; GCIPL, ganglion cell–inner plexiform layer; IOP, intraocular pressure; PPA, peripapillary atrophy; PICC, peripapillary intrachoroidal cavitation; mMNV, myopic macular neovascularization.

**Table 6:** Proposed minimum reporting set for studies of MON, GON, and overlap in highly myopic eyes.

Domain	Minimum Items to Report	Why It Matters
Myopia characterization	Spherical equivalent (diopters, D), axial length (mm), criteria for “high myopia”; presence and stage of myopic macular degeneration or other macular disease.	Definitions vary; macular comorbidity is a major confounder for OCT GCL and central VF interpretation.
ONH/parapapillary phenotype	Disc tilt and torsion descriptors; parapapillary atrophy/zones; presence of posterior staphyloma or parapapillary intrachoroidal cavitation (if assessed).	Phenotype strongly influences expected RNFL/GCL distribution and VF defect location in high myopia.
OCT protocol and quality	Device/model, scan protocol (RNFL circle diameter, macular scan), signal/quality metrics, and whether segmentation was manually verified or edited.	Artifacts and normative database mismatch are common; reproducibility and transparent reporting improve interpretability and comparability.
Functional testing	Perimetry strategy (24-2, 24-2C, 10-2), thresholding algorithm (e.g., SITA Standard vs. SITA Fast), reliability indices, number of baseline tests, and approach to learning effects.	Cross-sectional VF abnormalities are common in high myopia; longitudinal criteria depend on test strategy and reliability.
Progression definition	Explicit progression definition (event vs. trend), confirmation rules, and whether structure–function concordance was required.	Reduces misclassification between MON and true GON and enables meta-analytic synthesis.
Confounders and context	Macular pathology, cataract status/surgery, IOP/IOP-lowering therapy, systemic risk factors (e.g., sleep disorders), follow-up duration.	These factors modify risk and influence both structure and function over time.

Abbreviations: MON, myopic optic neuropathy; GON, glaucomatous optic neuropathy; OCT, optical coherence tomography; RNFL, retinal nerve fiber layer; GCL, ganglion cell layer; VF, visual field; IOP, intraocular pressure.

## Data Availability

No new data were created or analyzed in this study. Data sharing is not applicable to this article.

## Abbreviations

The following abbreviations are used in this manuscript:

BMO	Bruch’s membrane opening
GCL	Ganglion cell layer
GCIPL	Ganglion cell–inner plexiform layer
GON	Glaucomatous optic neuropathy
IOP	Intraocular pressure
mMNV	Myopic macular neovascularization
MON	Myopic optic neuropathy
OCT	Optical coherence tomography
OCTA	Optical coherence tomography angiography
ONH	Optic nerve head
PPA	Parapapillary atrophy
RNFL	Retinal nerve fiber layer
VF	Visual field
